# Reconstruction of SAXS Profiles from Protein Structures

**DOI:** 10.5936/csbj.201308006

**Published:** 2013-11-26

**Authors:** Daniel K. Putnam, Edward W. Lowe, Jens Meiler

**Affiliations:** a Center for Structural Biology; b Department of Biomedical Informatics; c Department of Chemistry, Vanderbilt University, Nashville, TN 37212, USA

**Keywords:** AXES, CRYSOL, DALAI, Debye formula, FOXS, PHAISTOS, SASTBX, SAXSTER, small angle X-ray scattering, spherical harmonics

## Abstract

Small angle X-ray scattering (SAXS) is used for low resolution structural characterization of proteins often in combination with other experimental techniques. After briefly reviewing the theory of SAXS we discuss computational methods based on 1) the Debye equation and 2) Spherical Harmonics to compute intensity profiles from a particular macromolecular structure. Further, we review how these formulas are parameterized for solvent density and hydration shell adjustment. Finally we introduce our solution to compute SAXS profiles utilizing GPU acceleration.

## Introduction

Small angle X-ray scattering (SAXS) is an experimental structural characterization method for rapid analysis of biological macromolecules in solution [1–6]. Because the samples do not need to be crystallized, they can be studied in different pH environments and concentrations leading to insightful structure-function relationships. The overall SAXS scattering profile is calculated by subtracting the scattering profile of the blank buffer solution from the profile of the sample dispersed in solution. SAXS data has been used to filter a set of protein models by comparing the SAXS profile of each model with the experimental SAXS profile [7, 8]. The SAXS profile has been incorporated as a term in the scoring function to obtain a protein model consistent with the experimental SAXS data [9]. An exciting feature in modern SAXS is identifying and modeling protein flexibility from an ensemble set of different conformers to fit experimental SAXS data [10, 11]. This requires a large library of starting conformers as input to the algorithm [12]. After a suitable library of conformers has been generated or found, the experimental SAXS data are used as a constraint in an algorithm to determine which combination of conformers optimally fit the data. The scattering intensity (I) is represented by a linear combination of the selected conformers. In this process the algorithm must decide 1) Which conformers to use and 2) How many conformers are required to accurately recreate the experimental SAXS profile. Critical to the success of this task are the underlying algorithms used to compute a SAXS profile from a proposed protein model. In this review we highlight different methods to accomplish this task. We recognize that these methods are not exhaustive of all methods, but represent a sampling of different approaches that provide insight to the process of computing SAXS profiles from atomic coordinates. For a more comprehensive review of small angle X-ray scattering theory we recommend several reviews [1–3, 13].

### X-ray Scattering Review

X-ray scattering is observed when differences in electron density exist in a given sample and X-rays generated from a source device pass through the sample. Although both coherent and incoherent scattering is possible, we will confine our considerations to coherent scattering because incoherent scattering is negligibly weak at very small angles [1]. Elastic (without energy change) electron scattering is influenced by all atomic orbitals. Because atomic orbitals have different shapes according to their atomic group, the X-ray scattering provides information on the structure of the target sample.

The scattering process occurs as electrons resonate with the frequency of the X-rays passing through the object. As the electrons resonate, they emit coherent secondary waves which undergo both constructive and destructive interference. Because of destructive interference, the superposition of waves with all possible phases will lead to zero scattering at a scattering angle of 2θ [1]. The scattering maximum I(0) will be theoretically observed at a scattering angle of zero where all waves are in phase. Because of the high intensity of the incident X-ray beam, a beam stop is placed between the detector and the beam to prevent it from distorting the scattering profile. I(0) must therefore be computed rather than experimentally observed.

To illustrate the scattering process, consider a linearly polarized monochromatic X-ray beam incident on a single electron with charge e and mass m. The periodic electric field of the incident X-ray produces a force on the electron (**F** = q_e_**E**) where **F** is the overall force the electron experiences, q_e_ is the charge of the electron and **E** is the electric field of the incident X-ray. This force causes the electron to oscillate with the same frequency as itself. The equations governing this behavior are shown below beginning with the electric field equation:

$$E\;=\;E_{0}e^{i(\mathrm{\omega t}-\delta )}$$

where **E** is the electric field, **E**_**0**_ is the maximum value of the electric field, ω is the frequency of oscillation of the wave-field, t is time, and δ is the phase constant.

**Figure 1:**
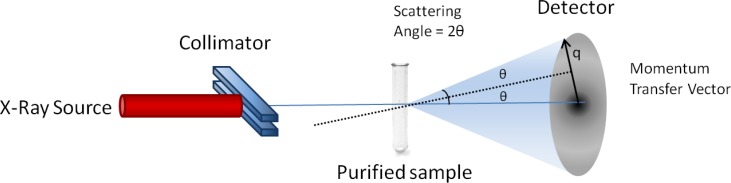
**SAXS Experimental Setup**. X-rays with a constant wavelength *λ* are first focused by the collimator and then pass through the purified sample in solution. A small fraction of the X-Rays scatter as they encounter electrons in the sample. The detector captures these scattered X-rays as intensity values. The final scattering profile is the difference between the profile of a blank buffer solution and a solution containing the purified sample.

By Newton's second law of motion we equate the two equations of force:

$$F=\mathrm{ma}=q_{e}E=q_{e}E_{0}e^{i(\mathrm{\omega t}-\delta )}$$

where m is the mass and **a** is the acceleration. The acceleration the electron experiences due to the periodic electric field is computed by dividing by the mass:

$$a=\frac{q_{e}}{m}E_{0}e^{i(\mathrm{\omega t}-\delta )}=A_{0}e^{i(\mathrm{\omega t}-\delta )}$$

where the amplitude **A**_**0**_ is:

$$A_{0}=\frac{q_{e}}{m}E_{0}$$

The electromagnetic radiation at a given distance with magnitude r from the charge q that experiences acceleration **a** has an electric field component:

$$\varepsilon =-\frac{q_{e}a\;\sin\;\alpha }{c^{2}r}$$

where c is the speed of light, r is the magnitude of the position vector, q_e_ is the charge, **a** is the acceleration and α is the angle between **a** and r. If the position of r is perpendicular to the incident beam (which is true for SAXS experiments) then α = 90° and sinα = 1. Combining this simplification with the electric field component and substituting **A**_**0**_ for **a**:

$$\varepsilon =-\frac{q_{e}A_{0}}{c^{2}r}=-\frac{q_{e}}{c^{2}r}\frac{q_{e}}{m}E_{0}=-\left(\frac{q_{e^{2}}}{mc^{2}}\right)\frac{E_{0}}{r}$$

Now imagine instead of a single electron, we have an electron cloud. As incident X-rays pass through an electron cloud with the origin at the center, most of them travel through the cloud without scattering, while a small fraction (< 1%) of the incident X-rays are scattered. This can be seen from the scattered to incident amplitude ratio:

$$F\frac{\varepsilon }{E_{0}}=-\left(\frac{e^{2}}{mc^{2}}\right)\frac{1}{r}=-\frac{r_{e}}{r}$$

where r_e_ is the constant Thomson scattering length and r is the distance from the object to the detector.

$$r_{e}=\frac{e^{2}}{mc^{2}}=\frac{1}{4\pi \in_{0}}\frac{q_{e}^{2}}{m_{e}c^{2}}=2.818\times 10^{-15}m$$

Because r_e_ is small, the scattered-to-incident amplitude ratio reveals that a single electron scatters a very small fraction of the incident X-rays. For example, at a sample to detector distance of three meters (typical for SAXS experiments), the amplitude ratio is:

$$\frac{r_{e}}{r}=\frac{2.818\times 10^{-15}m}{3\;m}\approx 10^{-15}$$

For an fuller description of the physics of X-ray scattering and the mathematics of waves we refer to the notes of Dr. Robert Blessing[14].

**Table 1:** Numerical values of critical constants in Thompson Scattering.

Name		Value
q_e_	Electron charge	1.602 x 10^-19^ C
m_e_	Electron rest mass	9.107 x 10^-31^ kg
c	Speed of Light	2.998 x 10^8^ m/s
ɛ_0_	Permittivity of free space	8.854 x 10^-12^ C^2^/Nm^2^

Because the scattered waves are coherent, the resulting amplitudes are added and the intensity is given by the absolute square of the amplitude [1]:

$$A=\sum_{i=1}^{n}A_{n};I=|A^{2}|$$

where A_n_ is the resulting amplitudes of all scattered waves and I is the scattering intensity. In Thompson elastic scattering all secondary waves have the same intensity and is given by [1]:

$$I_{s}(\theta )=I_{p}\cdot \left(\frac{e^{2}}{mc^{2}}\right)\cdot \frac{1}{r^{2}}\cdot \frac{1+\cos^{2}2\theta }{2}$$

where I_p_ is the primary intensity and I_s_ is the intensity of the secondary waves. The term e^2^/mc^2^ is the classical electron radius and, r is the distance from the object to the detector. For small angles the polarization factor (1 + cos^2^2ジ)/2 is approximately one leaving [1]:

$$I_{s}(\theta )=I_{p}\cdot \left(\frac{e^{2}}{mc^{2}}\right)\cdot \frac{1}{r^{2}}$$

### The Momentum Transfer vector

We will assume the amplitude and intensity of all secondary waves to be one for this discussion. With this framework, each secondary wave is represented by the complex function e^iφ^ where φ is the phase. Because the amplitude and intensity are one, all waves differ only by their phase. The phase of the scattered wave depends on the position of the oscillating electrons in space.

**Figure 2:**
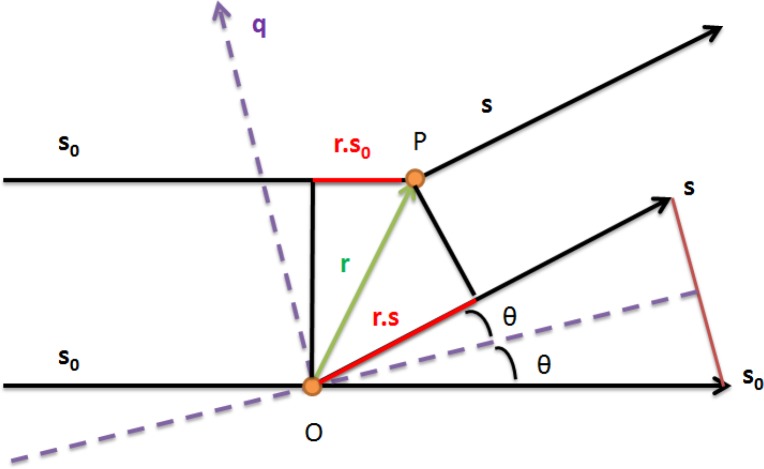
**X-Ray Scattering:** Adapted from Small Angle X-ray Scattering [1]. Incident (s_0_) and Scattered X-rays (s) with the derivation of the momentum transfer vector q.

The phase of the secondary waves is 2π/λ multiplied by the path difference between the scattered and incident waves. In the diagram, we let s_0_ represent the direction of the incident beam and we let s represent the direction of the scattered beam. The path difference of a point P, specified by **r**, against the origin O is: -**r**(**s**-**s**_**o**_). The phase is given by [1]:

$$\phi =-\frac{2\pi }{\lambda }r(s-s_{0});\phi =-qr$$

The term (**s**-**s**_**o**_) is symmetric to the incident and scattered beam with magnitude of 2sinθ. In this representation θ represents half the scattering angle. The momentum transfer vector **q** is independent of the distance to the detector and the wavelength (λ) and defines the scattering curve in reciprocal space with units of Å^-1^. The momentum transfer vector has the same direction as (**s**-**s**_**o**_) and the magnitude is given by substituting 2sinθ for (**s**-**s**_**o**_):

$$|q|=\frac{4\pi \cdot \sin(\theta )}{\lambda }$$

where 2θ is the scattering angle. We refer to q as the magnitude of the momentum transfer vector **q**. In the literature, this term has been defined multiple ways and one must be aware of the convention used. For example the symbols h and s have been used in place of q. Sometimes s is defined as s = (2sinθ)/λ with q = 2πs. Others define θ rather than 2θ as the scattering angle. In this review we use the convention for q shown above with 2θ as the scattering angle. Large interatomic distances contribute primarily to the scattered X-ray intensity at small scattering angles, whereas short interatomic distances primarily contribute to X-ray intensity at large scattering angles. The information content of a SAXS profile is small compared to other high resolution experimental techniques because the overall scattering profile represents the orientationally averaged contribution of all atoms in all orientations. The SAXS scattering curve contains information related to the overall shape of the molecule and is routinely used for the validation of structural models [15, 16].

### The Scattering Intensity Curve can be derived from the Electron Density Function

The term electron density is frequently used in the literature in the place of electron density difference or contrast. The electron density ρ is the number of electrons per unit volume. In SAXS experiments only the electron density difference ρ_2_ – ρ_1_ (ρ_2_ is the electron density of the sample, ρ_1_ is the electron density of the solvent) is measurable. If ρ_2_ = ρ_1_, then scattering is not observed because the waves scattered in any direction will cancel out. During a SAXS experiment the electron density of the buffer solution is subtracted from the density of the combined sample and buffer solution leaving the electron density of the sample without background solution.

The electron density function ρ(r) is defined in real space for non-negative values. It is a histogram of equivalent pairwise atomic distances in a given sample. Because of the solution subtraction, the electron density it is zero everywhere except for defined electron distances in the sample where identical distances add together.

**Figure 3:**
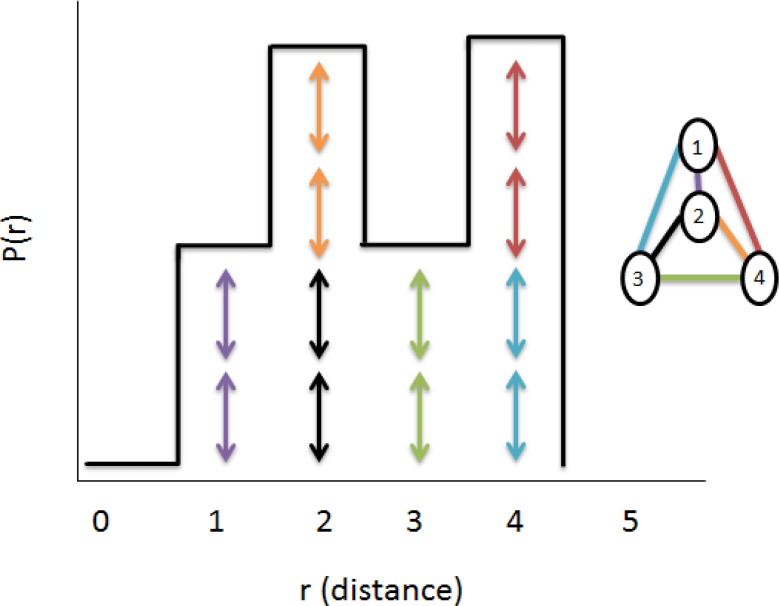
The pairwise distance distribution function adapted from X-ray solution scattering (SAXS) combined with crystallography and computation: defining accurate macromolecular structures, conformations and assemblies in solution [3]. Pair-wise distances between each atom are represented. The distances are symmetric and are represented twice by the double arrows. The P(r) function will be zero whenever a particular distance is not defined by the geometry of the sample.

If we have the distance distribution function then the scattering curve I(q) can be calculated by Fourier inversion[1]:

$$I(q)=4\pi \int_{0}^{\infty }\rho (r)\frac{\sin(qr)}{qr}\cdot dr$$

Likewise the distance distribution function ρ(r) can be calculated by Fourier inversion of the scattering curve [1]:

$$\rho (r)=\frac{1}{2\pi^{2}}\int_{0}^{\infty }I(q)\cdot qr\cdot \sin(qr)\cdot dq$$

Theoretical scattering curves can be computed for a model of a given shape and compared with experimental data using either the intensity calculation I(q) or the distance distribution function p(r). The distance distribution function allows the deduction of the largest particle dimension d_max_ and is the distance at which the p(r) drops to zero.

**Figure 4:**
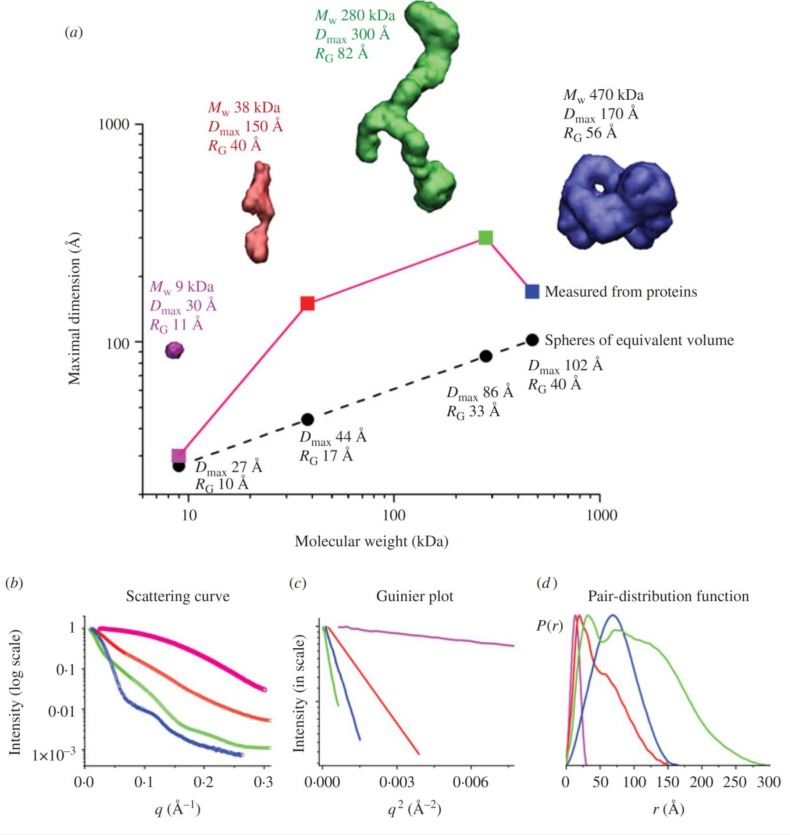
Originally from SAXS combined with crystallography and computation [3]. This figure depicts the experimental SAXS curves and parameters measured for *Pyrococcus furiosis* PF1282 rubredoxin (magenta), a ‘designed’ scaffoldin protein S4 (red), a ‘designed’ minicellulosome containing three catalytic subunits (green), and the DNA-dependent protein kinase (blue). (a) D_max_ of the scattering particle is a simple function of molecular weight for perfect spheres, but not for proteins that adopt different shapes. Envelopes correspond to *ab-initio* models calculated from experimental curves using GASBOR. (b) The experimental scattering curves for each protein show that the intensity of scattering falls more slowly for rebredoxin (R_G_ 11 Å ; magenta) than the minicellulosome (R_G_ 82 Å; green). (c) The linear region of the Guinier plot, from which R_G_ and I(0) can be derived, is a function of the R_G_. (d) Each protein has both a substantially different D_max_ as well as pair-distribution function, reflecting the different atomic arrangements.

### Debye Formula for computing scattering profiles from Atomic Coordinates

Proteins are built up from the arrangement of amino acids which are built up from the arrangement of atoms differing by side chain arrangement. Imagine a protein sample in a fixed orientation. The centers of mass of each atom may be designated by r_1_, r_2_, …, r_n_, and their amplitudes with respect to each mass center by f_1_, f_2_, …, f_n_. The total amplitude is [1]:

$$f_{protein}(q)=\sum_{j=1}^{N}f_{j}(q)\cdot e^{-iqrj}$$

where the additional phase factor describes the position of the atom and f_j_(q) is the amplitude. The intensity is the absolute square of the amplitude, averaged over all orientations:

$$I(q)=ff^{*}=‹\sum_{j=1}^{n}\sum_{k=1}^{n}f_{j}f_{k}^{*}\cdot e^{-iq(rj-rk)}›$$

When j=k the phase factor reduces to one. This situation represents the contribution to the intensity diffracted by the atoms alone. The situation j≠k represents the interference between the atoms, according to the relative distance (r_j_-r_k_). Each amplitude f has a phase:

$$f_{j}=║f_{j}║\cdot e^{i\phi_{j}}$$

Splitting the atomic diffraction (j=k) from the interference between atoms (j≠k) yields:

$$I(q)=\sum_{j=1}^{N}I_{j}(q)+2\cdot ‹\sum \sum_{j\neq k}^{n}|f_{j}||f_{k}|e^{i({\mathrm{qr}}_{\mathrm{jk}}+\phi_{k}-\phi_{j})}›$$

In SAXS experiments there is no fixed origin because particles are sampled in all orientations. The phase is dependent on a fixed origin. By averaging over all orientations and restricting atoms to be spherical, the phase vanishes, (φ_k_ - φ_j_) = 0 and f_j_ becomes independent of orientation. Furthermore, spherical averaging of all orientations is given by:

$$‹{\text{e}}^{{\text{iqr}}_{\mathrm{jk}}}›=\frac{\sin(q\cdot r_{\mathrm{ij}})}{q\cdot r_{\mathrm{ij}}}$$

This representation of the spherical averaging is known as the Debye factor [17]. The final Debye formula is:

$$I(q)=\sum_{j=1}^{N}I_{j}(q)+2\cdot \sum \sum_{j\neq k}^{n}f_{j}(q)f_{k}(q)\frac{\sin\;{\mathrm{qr}}_{\mathrm{jk}}}{{\mathrm{qr}}_{\mathrm{jk}}}$$

In this format the amplitudes f are calculated by computing the atomic structure factors. The atomic diffraction and interference between atom sums can be combined together to give the form of the Debye equation frequently cited in the literature:

$$I(q)=\sum_{i=1}^{N}\sum_{j=1}^{N}f_{i}(q)f_{j}(q)\frac{\sin(q\cdot r_{\mathrm{ij}})}{q\cdot r_{\mathrm{ij}}}$$

where r_ij_ = ∣ **r**_i_ –**r**_j_ ∣ are the x,y,z positions of atoms i and j. The Debye formula given above takes the atomic x,y,z coordinates as input and returns the intensity as a function of momentum transfer q. This double sum of all atoms in a given system for each computed q value has a computational cost of O(N^2^). The quadratic cost is a prohibitive barrier for atomic level application of the Debye formula for large systems (N > 10,000). In the case of structural refinement for SAXS, the scattering profile must be computed from all pairs of interactions with atoms in the molecule. In high-throughput applications the profile must be computed thousands of times, while in an iterative ensemble analysis, the profile must be computed hundreds of thousands of times. Because of the high computational cost, different methods have been developed to reduce the number of necessary calculations to compute intensity. Before we discuss the approximations to the Debye formula, we must first understand the structure factors f_i_(q) and f_j_(q).

### Structure Factors and Form Factors

The atomic form factor is a fundamental physical quantity in solid state physics. It is the Fourier transform of an electron distribution around a nucleus of a given atom and carries information on the electron wave function. The X-ray scattering power of a given atom will depend on the number of electrons it contains. As the number of electrons contained in an atom increases (higher atomic number), the scattering power increases. As the scattering angle increases, the scattering power decreases. A scattering angle of zero results in the maximum scattering factor for a particular atom which is equal to Z – the atomic number. The form factor approximations are based on the combination of relativistic Dirac-Slater wave functions and numerical Hartree-Fock wave functions [18–21]. These Hartree-Fock structure factors were computed from q = 0 to q = 1.5 at intervals of 0.01Å^-1^. For convenience, they were fit to a 5-gaussian (Cromer-Mann) analytic function:

$$f_{v,i}(q)=\sum_{i=1}^{4}a_{i}\cdot e^{-b_{i}{\left(\frac{q}{4\pi }\right)}^{2}}+c$$

where f_v_,_i_ (q) is the structure factor of a particular atom at a given q-value in vacuo. The constants a_1_, a_2_, a_3_, a_4_, b_1_, b_2_, b_3_, b_4_, and c are the Cromer-Mann coefficients for a given atom, and q is the momentum transfer in inverse angstroms. Tables for the Cromer-Mann coefficients are found in the International Tables for X-Ray Crystallography[22]. This approximation is valid in the q-ranges for SAXS scattering experiments from 0 to ≈ 0.33Å^-1^ [2, 3]. For larger q-ranges, a 6-gaussian approximation must be used which is valid from 0 to 6.0Å^-1^ [21].

In addition to the vacuo contribution to the form factors, the solvent makes a critical contribution to the overall scattered intensity. The solvent effect is considered by modeling the solvent as an electron gas with density equal to the average electron density of the solvent[23]. Taking the solvent effect into account, the overall structure factor of the atom is the combination of the structure factor representing the excluded solvent subtracted from the form factor for a given atom:

$${\text{F}}_{\text{i}}(\text{q})={\text{f}}_{\text{v},\text{i}}(\text{q})-{\text{f}}_{\text{s},\text{i}}(\text{q})$$

where fs,i is the structure factor of the hypothetical atom that represents the displaced solvent. The displaced solvent scattering term f_s,i_ is given by:

$${\text{f}}_{\text{s},\text{i}}(\text{q})=\rho {\text{V}}_{\text{i}}\text{e}-\frac{{\text{q}}^{2}{\text{V}}_{\text{i}}^{2/3}}{4\pi }$$

where ρ is the electron density of the solvent. For pure water this is 0.334e Å^-3^. V_i_ is the solvent volume V displaced by atom i and is calculated from the van der Waals radius of the atom.[23, 24]. The exponential term is the normalized Fourier transform of the Gaussian sphere. This sphere corresponds to the excluded volume around the atom.

The electron density surrounding the scattering body is calculated by computing the number of electrons per liter of solvent and then converting that to the number of electrons in a cubic angstrom. This excess electron density is then added to the density of pure water. Proteins have an electron density around 0.44e Å^-3^ [2]. The electron density of the solvent should maximize difference between itself and the electron density of the sample to maximize contrast in SAXS experiments. The derivation for the electron density of pure water with a density of 1g/mL is shown below:

$$\left[\frac{6.02\;*\;10^{23}H_{2}O\;\mathrm{Molecules}}{1\;\mathrm{mol}\;H_{2}O}\right]\;\left[\frac{10\;\mathrm{electrons}}{1\;H_{2}O\;\mathrm{Molecule}}\right]\;\left[\frac{1\;\mathrm{mol}\;H_{2}O}{18g}\right]\;\left[\frac{1g\;H_{2}O}{1{\mathrm{cm}}^{3}\;H_{2}O}\right]\;\left[\frac{1{\mathrm{cm}}^{3}\;H_{2}O}{10^{24}\;{Å}^{3}}\right]\approx \;0.334e\;{Å}^{-3}$$

Now that we have reviewed the theory of X-ray scattering and have an idea of the Debye equation with a costly double sum over all atoms, we are ready to review methods using the Debye equation designed to maximize accuracy while minimizing computation time.

### Fast approximation of the Debye Formula by Pantos and Bordas

In 1994, Pantos and Bordas used an approach to simulate SAXS patterns of large molecules by building models of closely packed spheres that are much larger than individual atoms thereby reducing N for the calculation. This was incorporated into the software package DALAI. They used the Debye formula to compute an intensity profile of the proposed model [25]:

$$I(q)=\sum_{j=1}^{N}I_{j}(q)+2\sum_{j=1}^{N}\sum_{k=1}^{N}F_{i}(q)F_{j}(q)\frac{\sin(q\cdot r_{\mathrm{ij}})}{q\cdot r_{\mathrm{ij}}},j\neq k$$

The first sum gives the intensity for spheres in isolation, while the double sum give the contributions from density-density correlations. To reduce the computational task in the double summations of the Debye equation, all spheres were given the same radius and mass density. The structure factor product F_i_(q)F_j_(q) is now constant for each value of q and can be pulled out of the double sum. The Debye formula becomes:

$$I(q)=\sum_{j=1}^{N}I_{j}(q)+2F^{2}(q)\sum_{j=1}^{N}\sum_{k=1}^{N}\frac{\sin(q\cdot r_{\mathrm{ij}})}{q\cdot r_{\mathrm{ij}}},j\neq k$$

At this point in the formulation, Pantos and Bordas have not compromised the accuracy of the calculation for the reduced sphere model. They moved the bulk of the computation to the initial state of the algorithm. The calculation of r_ij_ is still O(N^2^). To model large structures requiring a large number of spheres, they approximated pairwise distances between atoms. In this approach pair distances are grouped into a histogram of bin sizes based on the experimental data resolution. Without binning, the number of pairwise distance terms is equal to N(N-1)/2. In this method the distances were quantized to multiples of d_max_/100 where d_max_ is the maximum particle dimension. The resolution increases with decreasing bin size and decreases with increasing bin size. The resolution adjustment blurs the sampling grid by an undetectable amount in the resolution range of the simulation. The pair distance matrix of r_jk_ values are now a vector of distances weighted by the number of distances occurring in a given bin. The scattering formula becomes:

$$I(q)=\sum_{j=1}^{N}I_{j}(q)+2F^{2}(q)\sum_{k=1}^{\text{Nbins}}m(r_{k})\frac{\sin(q\cdot r_{k})}{q\cdot r_{k}}$$

where m(r_k_) is the bin population at pair distance r_k_ and the limits of the sum are the number of distance bins.

This method is valid when protein structures are modeled with multiple spheres of constant radii and mass density. When this condition is met, the structure factor calculation can be brought out of the double sum. The Debye calculation can then be binned leading to change of an O(N^2^) calculation to O(N). Prior to this calculation the pairwise distances must be pre-computed and binned which is still an O(N^2^) calculation. The speed increase by this algorithm is dependent on the number of spheres used to model the system. An advantage of this method is that the pairwise distance matrix must only be computed once and can then be reused during the course of analysis.

### Calculation of SAXS profiles with the Debye formula from coarse-grained protein models

In 2010, Stovgaard et al, used the Debye formula for calculating the scattering curve combined with a coarse-grained representation of protein structure to address the high computational cost [26]. This approach led to a significant speed-up in computational time when compared with the all atom calculation. In this approximation, amino acids were represented by two scattering bodies or dummy atoms – one representing the backbone, and the other representing the side chain. These dummy atoms were placed at the respective center of mass of the atomic group they represented. They had to estimate 21 form factor values for this approximation – one for alanine, one for glycine, one for the backbone, and 18 for the remaining side chains. They recreated these functions for each of the 21 form factors by binning the q-range into intervals of equal width (0.015 Å^-1^) and then computing a form factor estimate for each of the 21 form factor types in each of the q-bins. They sampled form factor values from a training set of 297 structures with lengths between 50 and 400 residues and calculated a form factor estimate from the centroid in each bin. The SAXS curves generated through the Debye formula with dummy atom form factors for 50 proteins were compared with SAXS curves generated for the same proteins through CRYSOL with high agreement.

This method is contingent upon the accuracy of the form factor estimates for the dummy atoms and relies on a training set of 297 proteins to represent amino acids in nature. Amino acid residues behave differently in different environments, and caution must be used to ensure the training set accurately represents the environment of the protein of interest. The authors state that two additional developments with this method are needed: 1) a proper description of the hydration layer and 2) a probabilistic description of the experimental errors associated with a SAXS experiment. This is currently under development in the PHAISTOS software package.

### The incorporation of the hydration layer into the Debye Formula via the form factor equations

In the same year that PHAISTOS was published, the Sali Lab published their approach to the Debye formula and made their web server FoXs publically available [27]. To account for the displaced solvent and hydration shell, the structure factor contribution for a given atom is given by:

$${\text{F}}_{\text{i}}(\text{q})={\text{f}}_{\text{v},\text{i}}(\text{q})-{\text{c}}_{1}{\text{f}}_{\text{s},\text{i}}(\text{q})+{\text{c}}_{2}{\text{S}}_{\text{i}}{\text{f}}_{\text{w},\text{i}}(\text{q})$$

where f_v,i_ (q) is the form factor of a particular atom at a given q-value without the effects of excluded volume and a water shell, and f_s,i_ is the structure factor for the excluded volume, and the last term is the structure factor of the hypothetical molecule that represents the displaced solvent. S_i_ is the solvent accessible surface area for a given heavy atom and f_w,i_ is the form factor of water. This approach is novel because it models the hydration shell as a function of the solvent accessible surface area of a given atom. The parameter c_1_ is used to adjust the electron density contrast while the parameter c_2_ is used to adjust the hydration shell thickness. The structure factor of water is given by the sum of all atomic form factors in water:

$$f_{w,i}(q)=2*f_{v,i}{\left(q\right)}_{\text{hydrogen}}+f_{v,i}{\left(q\right)}_{\text{oxygen}}$$

The computed profile was fit to a given experimental SAXS profile by minimizing the chi function with respect to c, c_1_, and c_2_:

$$\chi =\sqrt{\frac{1}{M}\sum_{i=1}^{M}{\left(\frac{I_{\text{exp}}(q_{i})-\mathrm{cl}(q_{i})}{\sigma (q_{i})}\right)}^{2}}$$

where I_exp_(q) and I(q) are the experimental and computed profiles, σ(q) is the experimental error of the measured profiles, M is the number of points in the profile, and c is the scale factor. The minimum value of chi was found by a computing c_1_ on the interval of [0.95, 1.12] and c_2_ on the interval of [0, 4.0] in steps of 0.005 and 0.1. Linear least squares minimization was performed to find the value of c that minimized chi for each c_1_ and c_2_ combination.

Similar to DALAI, FoXs has the structure factor calculation moved out of the double sum of the Debye formula. Instead of modeling uniform space filling spheres, they assumed an identical modulation of f_i_(q) for different atoms i:

$${\text{f}}_{\text{i}}(\text{q})={\text{f}}_{\text{i}}(0)\cdot \text{E}(\text{q})$$

where the modulation function E(q) is equal for all atoms. This approximation creates a system of different scattering masses but equal shape. The pairwise distance distribution function represents population at a given distance r and is given in this approximation as:

$$\rho (r)=\sum_{i,j}f_{i}(0)f_{j}(0)\cdot \delta (r-d_{\mathrm{ij}})$$

where *δ*(r-d_ij_) is the Dirac-Delta distribution and r is a given pairwise distance. In this representation, only the form factor with a constant q = 0 is considered, which reduces the value to the atomic number Z of the given value. The intensity is given by:

$$I(q)=E^{2}(q)\cdot \int_{0}^{\infty }\rho (r)\frac{\sin(\mathrm{qr})}{\mathrm{qr}}\mathrm{dr}$$

The modulation function E^2^(q) is parameterized as:

$${\text{E}}^{2}(\text{q})={\text{e}}^{\left(-\text{b}*{\text{q}}^{2}\right)}$$

The parameter b was determined by computing the SAXS profile with the original Debye formula using the non-approximated form factors and then computing the SAXS profile with the approximated form factors and initial guess of the b parameter. The parameter b = 0.23±0.01 Å^-1^ was chosen to minimize the difference between both profiles from 30 random protein structures from the Protein Data Bank. This approximation typically speeds to calculation of the Debye formula by two orders of magnitude.

### The explicit incorporation of the hydration layer into the Debye Formula

In 2011, the Zhang lab at the University of Michigan introduced SAXSTER, an online tool to improve protein template recognition by using SAXS profiles[28]. In their approach they also simulate the SAXS intensity profile according to the Debye equation. Instead of summing over all atoms, they sum over all atoms plus the explicit water atoms. The equation is:

$$I(q)=\sum_{i=1}^{N+W}\sum_{j=1}^{N+W}F_{i}(q)F_{j}(q)\frac{\sin(q\cdot r_{\mathrm{ij}})}{q\cdot r_{\mathrm{ij}}}$$

where W is the number of “dummy” water molecules around the protein representing the hydration shell. The initial structure factor equations are identical to equations previously shown. To account for the explicit water molecules around the model, they started from a face-centered cubic (FCC) lattice system with edge length L_cell_. Each point in the lattice represents a water molecule. The overall structure factor is given by subtracting the excluded solvent from the atomic form factor and adding the explicit water contribution from the lattice. The protein structure is projected onto the FCC system and only water molecules in the range of 3.5-6.5 Å to any Cα atoms are kept. The density of the water molecules in the lattice system is defined by:

$$\rho_{\text{FCC}}=\frac{N_{\text{FCC}}}{V_{\text{FCC}}}=\frac{4k^{3}}{L^{3}}$$

where N is the number of points in the FCC lattice system, V is the volume of the system, k is the number of unit cells in the x,y,z directions and L = k * L_cell_. L represents the maximum length for each direction. In a FCC lattice system, the water contribution from each corner of the cubic cell is 1/8 and the contribution from each face is 1/2. There are eight corners and six sides yielding an effective water contribution of four (8(1/8) + 6(1/2)). Each water molecule consists of 10 electrons yielding 40 (water contribution of four * 10 electrons) electrons per cubic cell. The number of excess electrons per volume in the hydration shell relative to the bulk water is:

$$\delta \rho =\frac{40\;\text{electrons}}{L_{\text{cell}}^{3}}=\rho_{\text{shell}}-\rho_{\text{bulk}}$$

The thickness of the hydration shell is thus controlled by the edge Length of the FCC system. The threading-based models are composed of α-carbons only and the SAXS computations are given by:

$$I(q)=\sum_{i=1}^{N+W}\sum_{j=1}^{N+W}F_{\text{eff}}^{i}(q)F_{\text{eff}}^{i}(q)\frac{\sin(q\cdot r_{\mathrm{ij}})}{q\cdot r_{\mathrm{ij}}}$$

$$\rho (r)=\sum_{i=1}^{N+W}\sum_{j=1}^{N+W}F_{\text{eff}}^{i}(q=0)F_{\text{eff}}^{j}(q=0)\delta (r-r_{\mathrm{ij}})$$

This form of the ρ(r) function is very similar to FoXs. The difference is that the water molecules are explicitly summed over. In the approximation, a new structure factor must be derived to represent the α-carbons:

$$F_{\text{eff}}(q)={‹\sum_{i=1}^{N+W}\sum_{j=1}^{N+W}F_{i}(q)F_{j}(q)\frac{\sin(q\cdot r_{\mathrm{ij}})}{q\cdot r_{\mathrm{ij}}}›}^{1/2}$$

where 〈…〉 denotes the average over all residues of the same type calculated from 200 randomly selected PDB structures. The term f(q) is computed by the initial structure factor equations previously shown. This procedure produces 20 effective structure factors for each amino acid type. In the case of water, its scattering factor is calculated by the modified Debye equation with n = 3, r_ij_ = 0 and F_i_(q) being the vacuum form factors for either hydrogen or oxygen.

### Spherical Harmonics - A second widely used approach to address the computational cost of SAXS profile reconstruction

In the methods previously described, the orientational averaging of the scattered waves was computed analytically using the Debye relation [17]:

$$‹e^{\mathrm{iqr}}›=\frac{\sin(q\cdot r)}{q\cdot r}$$

Instead of analytically computing the orientational averaging, another method is to use a mathematical representation of the scattering body (or protein) that uses the rotational properties of spherical tensors. In this formulation the scattering body is expanded in terms of an infinite series of spherical harmonics. The orthogonality properties of the basis functions simplify the averaging of the harmonic series from which an overall scattering intensity can be computed. These basis functions are built from spherical Bessel functions, and normalized spherical harmonics of degree m and order L. This approach reduces the computational complexity from O(N^2^) to O(N).

The scattering amplitude in vacuo of a particle with N atoms is:

$$A_{\text{vacuo}}(q)=\sum_{j=1}^{N}f_{j}(q)e^{{\text{iqr}}_{j}}$$

where r_j_ = (r_j_,ω_j_) = (r_j_,φ_j_,φ_j_) and f_j_ is the corresponding atomic form factors. Spherical averaging is simplified by multipole expansion [29]:

$$e^{\text{iqr}}=4\pi \sum_{L=0}^{L_{\text{max}}}\sum_{m=-L}^{L}i^{L}j_{L}(\text{qr})Y_{\text{Lm}}^{*}(\omega )Y_{\text{Lm}}(\Omega )$$

where j_L_(qr) are the spherical Bessel functions of order L and Y_Lm_(Ω) are the spherical harmonics of order (L,m). The angular symmetry of Y_Lm_ is related to the symmetry of the multipoles: L=0 (monopole) L=1 (dipole), L=2(quadrupole), etc. Substituting the multipole expansion with spherical harmonics for the exponential term yields:

$$A_{\text{vacuo}}(q)=\sum_{L=0}^{L_{\text{max}}}\sum_{m=-L}^{L}4\pi i^{L}Y_{\text{lm}}(\Omega )\sum_{j=1}^{N}f_{j}(q)j_{L}(qr_{j})Y_{\text{Lm}}^{*}(\omega_{j})$$

where (r_j_,ω_j_) are the polar coordinates of the j_th_ atom. The partial amplitudes can be separated from the proceeding equation:

$$A_{\text{vacuo}}(q)=\sum_{L=0}^{L_{\text{max}}}\sum_{m=-L}^{L}A_{\text{LM}}(q)Y_{Lm}(\Omega )$$

where A_Lm_(q) are the partial amplitudes and are given by:

$$A_{\text{Lm}}(q)=4\pi i^{L}\sum_{j=1}^{N}f_{j}(q)j_{L}(qr_{j})Y_{Lm}^{*}(\omega_{j})$$

Because of the orthogonality properties of spherical harmonics, the cross terms cancel and the intensity calculation is reduced to [30]:

$$I_{\text{vacuo}}(q)=\int |A_{\text{Lm}}(q){|}^{2}\text{ds}(u)=\sum_{L=0}^{L_{\text{max}}}\sum_{m=-L}^{L}|A_{\text{Lm}}(q){|}^{2}$$

The huge advantage of spherical harmonics is that the complexity is reduced from O(N^2^) to O(N). The integrand for averaging over the sphere in the proceeding equation is approximated by an L = O(qD) band limited function in a spherical harmonic basis where q is the momentum transfer vector and D is the maximum dimension of the sample. It is insufficient to use L smaller than qD/2 because any value less than this violate Nyquist Shannon sampling[31] for periodic functions. At least L^2^ = O(q^2^D^2^) sampling points are needed to provide an accurate integration of bandwidth L. Any index above L does not improve the fit for a given q_max_, while any index below L will result in systematic errors in the calculation[32].

### CRYSOL – The incorporation of the hydration shell using spherical harmonics with multipole expansion to compute SAXS profiles from atomic coordinates

By the early 1990s there were many studies showing the importance of modeling the water molecules surrounding a given macromolecule when recreating SAXS profiles from atomic coordinates. For example, Grossman et. al compared experimental SAXS profiles with SAXS profiles computed from different configurations of dimers, trimers, and tetramers. They optimized the agreement between experimental and simulated scattering profiles by placing solvent molecules on a diamond-shaped grid surrounding the structure[33]. In their results, the computed SAXS profile with the best fit to the experimental SAXS profile consisted of a solvent shell of 716 water oxygens up to a maximum distance of 3.15 Å from the protein surface. Their results suggested that the water shell very close to the surface of a protein differs in electron density from the remaining bulk water and thus contributes to x-ray scattering.

**Figure 5:**
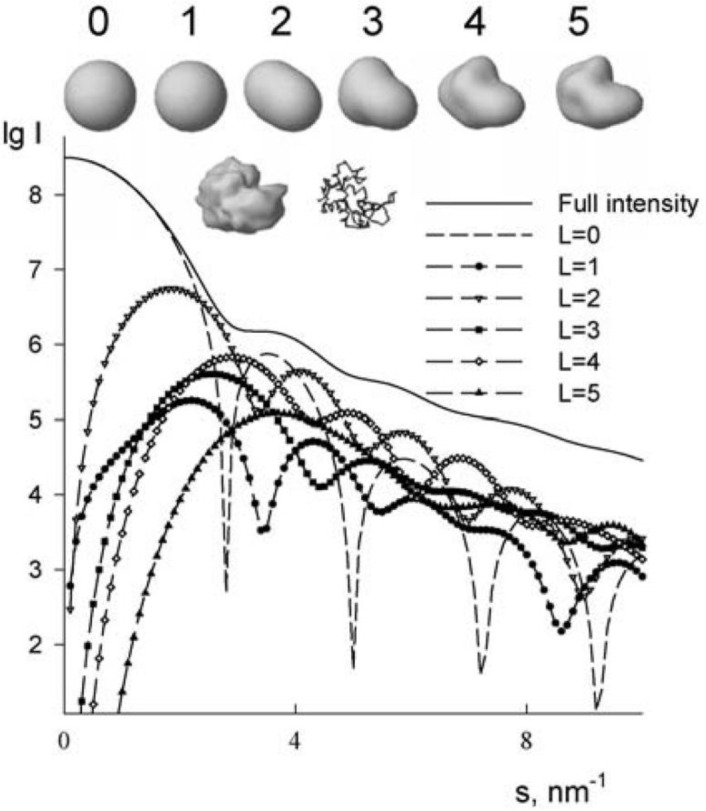
Originally from Models, structures, interactions and scattering [2]. Accuracy shape representations using spherical harmonics. Top row: surface representations of truncated envelope functions of lysozyme. Second row: high-resolution envelope functions and C_α_ trace of the protein. The shape scattering intensity from lysozyme is shown along with the contributions from different multipoles.

In 1995 Svergun et. al released CRYSOL – a program to compute SAXS intensity profiles from atomic coordinates while considering the hydration shell surrounding the target sample[24]. There were lingering questions concerning the true cause of the electron density contrast conditions surrounding a sample in solution. Was the density contrast caused by a water layer or could the contrast be explained by side chains moving freely on the protein surface? Three years later in 1998 Svergun et al. confirmed in a combined X-ray and neutron scattering study that the differing electron contrast conditions were more likely caused by a denser hydration shell rather than a higher mobility of the side-chains on the protein surface[34]. Water modeling is critical to the correct interpretation of SAXS profiles and computational methods are under development today to improve chemistry constraints, improve geometric constraints (surface curvature), and incorporate experimental data from high-angle SAXS[35].

Currently, popular approaches for modeling the hydration shell are to: 1) place water molecules on the surface of the protein, 2) simulate the solvation shell by surrounding the protein with a continuous outer envelope, 3) simulate the solvation shell and excluded volume by computing a modified scattering factor.

CRYSOL employed the second approach to model the hydration shell and extended the multipole expansion and spherical harmonics formulation to handle not only the vacuo scattering, but the excluded volume and hydration shell.[9]

In this formulation the intensity is given by:

$$I(q)=‹|A_{a}(q)-\rho_{0}A_{c}(q)+\delta \rho A_{b}(q){|}^{2}{›}_{\Omega }$$

where A_a_(**q**) is the in vacuo scattering, A_c_(**q**) is the excluded volume scattering and A_b_(**q**) is the border layer scattering, δρ = *ρ*_b_ – *ρ*_0_, where *ρ*_0_ is the average scattering density of the solvent surrounding the particle and *ρ*_b_ is the average scattering density of the border layer around the particle with thickness ▵. 〈 〉_Ω_stands for the average over all particle orientations and Ω is the solid angle in reciprocal space, **q** = (q, Ω). Each of the three amplitudes is represented via its multipole components. Because of the orthogonal properties of the spherical harmonics, all cross terms cancel in the average over Ω, leading to:

$$I(q)=\sum_{l=0}^{L}\sum_{m=-l}^{l}|A_{lm}(q)-\rho_{0}C_{lm}(q)+\delta \rho B_{\text{lm}}(q){|}^{2}$$

The value L defines the resolution of the particle. This approach works best with shapes that can be described using spherical harmonics which include most globular and extended proteins. Spherical harmonics is less adept at handling shapes that contain internal cavities such as shells and donuts.[24] Additionally this method uses by default a harmonic order of 15, with a maximum value of 50. This gives the method a complexity of O(MN) with M=q^2^D^2^. This can lead to errors when a harmonic order greater than 50 is necessary based on the size of the protein and desired q_max_.

In CRYSOL there are several adjustable parameters used when calculating predicted data that best match the experimental curve. These parameters are: the effective atomic radii multiplier which scales the solvent volume displaced by each atom (v_i_), the electron density contrast of the surface solvent layer (c_2_) and the total displaced solvent volume (c_1_), approximately equal to the variation of the electron density of the displaced solvent relative to bulk water. The need for adjustable parameters in CRYSOL becomes clear when studying SAXS profile reproducibility for distinct samples of the same protein on different instruments. The characteristic features of the experimental scattering profiles are conserved between experiments, but the experimental variation of the scattered intensity at higher q-values depends on the extrapolated intensity at I(0)[36]. Because of the beamstop in a SAXS experiment, I(0) cannot be directly observed. One method to extrapolate this value is to compute the slope of the intensity profile in the initial linear region of the scattering profile (the Guinier region) and extrapolate to the y-intercept. The adjustable parameters in CRYSOL absorb this variability by changing the level of the higher-q features of the predicted data relative to the low-q intensities.

### Extension of CRYSOL to improve accuracy

Fifteen years after the introduction of the original CRYSOL program, Alexander Grishaev, Liang Guo, Thomas Irving and Ad Bax introduced AXES in 2010 – a program for fitting SAXS data to macromolecular structure and ensembles of structures[36]. The program AXES was designed to be more discriminating than CRYSOL when evaluating poorly or incorrectly modeled protein structures. On a set of small well-studied proteins that had X-ray crystallography and solution NMR data they reported an improvement in fit by 10-50% by χ score. This set was comprised of four proteins – hen egg white lysozyme, cytochrome c, the B3 domain of protein G (GB3) and ubiquitin.

They reformulated the approach to fitting SAXS data by explicitly taking into account the sources of experimental data variability:

$$I_{\text{exp}}(q)=I_{\text{sample}}(q)-\alpha I_{\text{buffer}}+c$$

where α accounts for the uncertainty in the measurements and c accounts for the variability of the detector and X-ray fluorescence. These uncertainties appear responsible for the systematic difference between repeated experimental data sets. Taking these uncertainties into account, the computed scattering intensity is:

$$I(q)={‹{‹‹|A_{a}(q)-\rho_{0}A_{c}(q)+\delta \rho A_{b}(q){|}^{2}{›}_{\Omega }›}_{\text{solv}}›}_{\text{ens}}$$

where Ω is the average taken over a discrete set of molecular orientations relative to the incident beam, solv is the average taken over the displaced and surface water sets, and ens is the average over the ensemble of macromolecular structures. The program AXES models the hydration shell directly by using explicit water molecules from a pre-equilibrated water box.

Using this approach they tested how well they could discriminate different models of the same protein. They generated 2000 models of GB3 using Rosetta and fit the experimental SAXS data to all of the models using both CRYSOL and AXES. The CRYSOL fits yielded χ values that were much lower for poor models (models with a high RMSD relative to the native structure) than the native structure. This behavior is indicative of overfitting. Using AXES, they did not observe significantly better fits for the poor Rosetta models. Furthermore, when provided chemical shift guided Rosetta models with the correct fold, AXES correctly assigned higher χ values to non-native structures.

The cost of this higher precision comes at the price of computation time. AXES is more than an order of magnitude slower than CRYSOL due to the averaging of the scattering amplitudes of the displaced and surface solvent sets over 20 different configurations. Among these configurations are: 6 elementary scattering functions averaged over angular orientations, macromolecular conformers, and molecular solvent configurations for a given electron density contrast of the surface solvent layer. Currently several avenues for computation speedup are under development.

### The use of Zernike polynomials to compute SAXS scattering profiles

We previously mentioned three popular approaches for treating the hydration shell and excluded solvent. They were: 1) to place water molecules on the surface of the protein and compute scattering profiles with explicit water molecules, 2) simulate the solvation shell by surrounding the protein with a continuous outer envelope, 3) simulate the solvation shell and excluded volume by computing a modified scattering factor. The drawback to the first approach is the computational cost to construct the explicit solvent model. The drawback of the second approach occurs for proteins containing cavities. Assuming a uniform layer around a cavity or hole will introduce artificial areas without any electron density. The drawback of the third approach is the appearance of non-uniformities in the electron density by overlapping dummy atoms.

In 2012, Liu et al proposed a new method to address the limitations of excluded solvent and hydration shell modeling[30]. In their approach they parameterized the Fourier transform of the electron density distribution function p(r) by a Zernike polynomial expansion with spherical harmonics. Zernike polynomials are orthogonal functions on the unit ball. They reformulated the SAXS intensity calculation as:

$$I(q)=16\pi^{2}\sum_{n=0}^{\infty }\sum_{n^{\prime }=0}^{\infty }b_{n}(qr_{\text{max}})b_{n^{\prime }}(qr_{\text{max}})F_{nn^{\prime }}$$

$$b_{n}(qr_{\text{max}})=\frac{j_{n}(qr_{\text{max}})+j_{n+2}(qr_{\text{max}})}{2n+3}$$

where j_n_ is the spherical Bessel function of order n.

$$F_{nn^{\prime }}=\sum_{l=0}^{n}k_{nn^{\prime }l}\sum_{m=-l}^{l}c_{\text{nlm}}c_{n^{\prime }\text{lm}}^{*}$$

where c_nlm_ is the Zernike moments from three-dimensional objects and k_nn'l_ is either a positive or negative coefficient given by:

$$k_{nn^{\prime }l}={\left(-1\right)}^{\frac{n+n^{\prime }}{2-l}}$$

The Zernike moments are computed by a linear combination of the geometric moments of the object:

$$c_{\text{nlm}}=\frac{3}{4\pi }\sum_{r+s+t\leq n}\bar{\chi_{\text{nlm}}^{\text{rst}}}M_{\text{rst}}$$

where M_rst_ is the geometric moment and $\chi_{nlm}^{rst}$ are the coefficients. The procedure to compute the coefficients are given by the Novotni and Klein algorithm [37].

$$M_{\text{rst}}=\int_{|r|\leq 1}\rho (r)x^{r}y^{r}z^{r}\text{dr}$$

The geometric moments are computed from a scattering object that has been segmented into a series of small volume cubes called voxels. Voxels are used in 3D graphics for the visualization and analysis of medical and scientific data. In this case the voxelization process maps electron density from the scatterer (or protein) into voxels from which the geometric moments can be computed. From this process, multiple sets of voxels are created: 1) **P** – the set of non-zero electron density voxels, 2) **S**+**B** – the set of voxels representing the excluded solvent and surface bound solvent, and 3) **S** – the set of voxels representing the excluded solvent.

The Zernike moments of all three voxelized objects are combined by a weighted sum to produce one set of Zernike moments from which the scattering intensity is computed. The computational complexity of this algorithm is O(N), but prior to computation, the voxelized object must be created in a preprocessing step.

The advantage of the Zernike expansion method is that it can model holes or cavities of structures that spherical harmonics traditionally has difficulty with. This approach also incorporates all solvent-accessible surfaces into the overall scattering profile. When compared on a set of ten experimental proteins with high resolution crystal structures, this method had similar results with the spherical harmonic expansion method. This method offers an extension to spherical harmonic expansion methods that may improve the fit to experimental data by improved hydration shell and excluded volume treatment of structures with cavities or holes. It is included in the SASTBX software package.

**Table:** Summary of Techniques

Year	Method	Complexity	
		Big O	M
1994	**DALAI** ( Debye with binned pairwise distance) $\text{I}(\text{q})=\sum_{\text{j}=1}^{\text{N}}{\text{I}}_{\text{j}}(\text{q})+2{\text{F}}^{2}(\text{q})\sum_{\text{k}=1}^{\text{Nbins}}\text{m}({\text{r}}_{\text{k}})\frac{\sin(\text{q}\cdot {\text{r}}_{\text{k}})}{\text{q}\cdot {\text{r}}_{\text{k}}}$	O(N^2^)	-
1995	**CRYSOL** (Multipole expansion and spherical harmonics) $\sum_{\text{L}=0}^{{\text{L}}_{\text{max}}}|\sum_{\text{m}=-\text{L}}^{\text{L}}{|4\pi {\text{i}}^{\text{L}}\sum_{\text{j}=1}^{\text{N}}{\text{f}}_{\text{j}}(\text{q}){\text{j}}_{\text{L}}(\text{q}{\text{r}}_{\text{j}}){\text{Y}}_{\text{Lm}}^{*}(\omega_{\text{j}})|}^{2}$	O(MN)^35^	(q^2^D^2^)^32^
2010	**PHAISTOS** (Debye with Bayesian modeling of form factor) $\text{I}(\text{q})=\sum_{\text{i}=1}^{\text{N}}\sum_{\text{j}=1}^{\text{N}}{\text{f}}_{\text{i}}(\text{q}){\text{f}}_{\text{j}}(\text{q})\frac{\sin(\text{q}\cdot {\text{r}}_{\text{ij}})}{q\cdot r_{\text{ij}}}$	$\text{O}\left({\left[\frac{M}{k}\right]}^{2}\right)$ [38]	M: number of atoms in the structure K: number of atoms described by a dummy body. K_ave_ = 4.24
2010	**FOXS** (Debye with approximated structure factor) $\text{I}(\text{q})=\sum_{\text{i}=1}^{\text{N}}\sum_{\text{j}=1}^{\text{N}}{\text{f}}_{\text{i}}(\text{q}){\text{f}}_{\text{j}}(\text{q})\frac{\sin(\text{q}\cdot {\text{r}}_{\text{ij}})}{\text{q}\cdot {\text{r}}_{\text{ij}}}$	O(N^2^)^35^	-
2010	**AXES** (multiple averaging with spherical harmonics and explicit water molecules) $‹‹‹|{\text{A}}_{\text{a}}(\text{q})-{\text{ρ}}_{0}{\text{A}}_{\text{c}}(\text{q})+\delta \rho {\text{A}}_{\text{b}}(\text{q}){|}^{2}{›}_{\Omega }{›}_{\text{solv}}{›}_{\text{ens}}$	O(MN)^35^	M: number of spherical grid points
2011	**SAXSTER** (Debye with explicit water molecules) $\text{I}(\text{q})=\sum_{\text{i}=1}^{\text{N}+\text{W}}\sum_{\text{j}=1}^{\text{N}+\text{W}}{\text{f}}_{\text{i}}(\text{q}){\text{f}}_{\text{j}}(\text{q})\frac{\sin(\text{q}\cdot {\text{r}}_{\text{ij}})}{\text{q}\cdot {\text{r}}_{\text{ij}}}$	O([N + W]^2^)	-
2012	**SASTBX** (3D Zernicke polynomials) ${|\sum_{\text{n}=0}^{{\text{n}}_{\text{max}}}\sum_{\text{l}=0}^{\text{n}}\sum_{\text{m}=-\text{l}}^{\text{l}}{\text{i}}^{\text{l}}{\left(-1\right)}^{(\text{n}-1)/2}{\text{c}}_{\text{nlm}}Y_{\text{lm}}^{*}({\text{w}}_{\text{q}}){\text{b}}_{\text{n}}(\text{q})|}^{2}$	O(MN)^35^	(N_max_ + 1)^2^

### Recent developments for SAXS profile reconstruction using GPU acceleration

In 2012, the SAXS algorithm in PHAISTOS was accelerated using general purpose graphical processing units (GPGPUs)[38]. This method utilizes Bayesian probability statistics to compute the form factors in the Debye equation for protein models built from either one or two scattering bodies. The speed up using GPU's was measured from protein sizes ranging from 64 to 8192 scattering bodies. They reported a 16x speed up for proteins with 64 scattering bodies. As the proteins increased in size the speed up increased to a maximum speed up of 394x for proteins with 8192 scattering bodies.

Because of the uncertainty introduced into the accuracy of the Debye equation by approximation methods, we devised a method to compute the intensity directly without approximating structure factor calculations (unpublished). Furthermore, we model the hydration shell as a function of the solvent accessible surface area of a given atom analogous to FoXs. Our method BCL::SAXS offsets the high computational cost of the Debye formula by simultaneously computing multiple pieces of the equation using the parallel architecture of graphical processing units (GPUs). The Debye formula can be framed as an NxN square matrix of N-atom rows by N-atom columns where N is the number of atoms in a given protein. The pairwise Euclidean distances (r_ij_) are calculated from the upper triangle of the matrix. The diagonal is set to zero and the lower triangle is a symmetric mirror of the upper triangle. Each GPU thread computes a partial Debye sum.

$$I_{\text{partial}}(q)=\sum_{i=1}^{N}F_{i}(q)F_{j}(q)\frac{\sin(q\cdot r_{\text{ij}})}{q\cdot r_{\text{ij}}}$$

This results into a matrix of q rows by N-atom columns where q is the momentum transfer and N is the total number of atoms. These partial values are summed across each column to complete the intensity computation:

$$I_{\text{total}}(q)=\sum_{i=1}^{N}I_{\text{partial},n}$$

This approach removes the uncertainty introduced by structure factor approximation while maintaining the efficiency of methods with structure factor approximations. The speed up using GPU's was measured from protein sizes ranging from 1832 atoms (PDB ID: 1O26) atoms to 91,846 (PDB ID: 1VSZ). Using a GTX680 GPU card, we observed a 5x speed up for the smaller protein (1O26). For the largest protein in our set (1VSZ) we observed a speed up of 1707x for protein 1VSZ using the same graphics card. By leveraging GPU's, we absorb the O(N^2^) cost while achieving substantial reduction in computation time without sacrificing accuracy by introducing approximations to the Debye formula.

## Conclusion

In this review we focused on proteins as a scattering body, but RNA and DNA can be studied as well using SAXS. These algorithms represent a sampling of methods for SAXS profile reconstruction and are not representative of all the approaches that exist. Another approach that expands these ideas was published in 2012. In this work, Gumerov et. al proposed a Hierarchal algorithm based on a fast multipole method (FMM) to compute SAXS profiles[32]. For a review of timing and accuracy for protein of varying sizes and shapes with either spherical harmonic or Debye implementations we refer to their work. In each of the algorithms presented, there was a trade-off between speed and accuracy. In order to use the Debye formula for protein structure analysis, approximations were made to the equation to move terms out of the double sum. The uncertainty introduced by this approach is a subject for further study. In order to model with spherical harmonics, the correct harmonic order must be set and the shape complexity of the scattering body must be considered. We expect that more algorithms in the near future will take advantage of the parallelizable form of the Debye equation and use GPU acceleration to obtain the necessary computational speed without the uncertainty introduced by structure factor approximation and momentum transfer binning.

Furthermore, to standardize testing of SAXS algorithms we echo the suggestion of Rambo and Tainer and believe a reference dataset should be created with experimental SAXS profiles and PDB models[35]. This dataset would be comprised of proteins of varying sizes and shapes and folds. All new and existing methods should be benchmarked against this set to identify strengths and weakness of any given algorithm.
